# Cognitive, social, and behavioral effects of music and motor intervention in children with autism spectrum disorder: the role of time of day

**DOI:** 10.3389/fped.2025.1683930

**Published:** 2025-10-30

**Authors:** Chayma Kanzari, Aymen Hawani, Bassem Mkaouer, Maher Mrayeh, Santo Marsigliante, Antonella Muscella

**Affiliations:** ^1^ High Institute of Sport and Physical Education of Kef, University of Jendouba, El Kef, Tunisia; ^2^ Higher Institute of Sport and Physical Education (Ksar Saïd), University of Manouba, Manouba, Tunisia; ^3^ Physical Activity, Sport and Health, Research Unit (UR18JS01), National Observatory of Sport, Tunis, Tunisia; ^4^ Department of Biological and Environmental Science and Technologies (DiSTeBA), University of Salento, Lecce, Italy

**Keywords:** autism spectrum disorder (ASD), time of day, music, movement, physical activity, intervention, cognitive functions, stereotypical behavior

## Abstract

This study aimed to determine the effect of 12 weeks of specific training (combining movement and music intervention) on children with autism spectrum disorders (ASD), specifically by comparing the time of day (morning vs. afternoon) with cognitive functions, mood, and social integration. Thirty children (19 males, 11 females; mean age 7.8 ± 1.27 years) were randomly assigned to two groups: an Intervention Group and a Control Group (CG). The intervention protocol consisted of combined sessions of training, music, and motor activities, administered in a counterbalanced order: one session in the morning (9:00–9:45 a.m.) and one in the afternoon (4:00–4:45 p.m.). The control group continued their regular physical activity. Children were assessed at baseline and post-intervention for cognitive functions (Trail Making Test), maladaptive behaviors (RCS), and enjoyment (PACES). A repeated-measures ANOVA was used to analyze the interaction between the music and movement intervention and time of day. Results showed a significant increase in enjoyment in both experimental groups (morning and afternoon) compared to the control group (*p* < 0.001), with no significant difference between the morning and afternoon groups (*p* = 0.743). After 12 weeks, the experimental groups showed significant improvements in both stereotypical behaviors (*p* < 0.001) and cognitive functions (*p* < 0.001). However, the time of day did not significantly influence these improvements (*p* = 0.133 for stereotypical behaviors and *p* = 0.681 for cognitive functions). Significant improvements were observed in affective/emotional (*p* < 0.001) and motor control behaviors (*p* < 0.001), which partially reflect reductions in maladaptive behaviors. However, specific measures of social engagement did not show statistically significant changes (*p* > 0.05). Our study found no effect of time of day on the outcomes for children with autism spectrum disorders.

## 1 Introduction

Autism spectrum disorder (ASD) is a neurodevelopmental condition characterized by persistent deficits in language (1), communication (2), and socialization (3), as well as the presence of stereotyped or repetitive movements (4).

Children diagnosed with autism tend to have a higher rate of movement disorders, social interaction, and communication, difficulty switching between activities, focusing on details, and unusual reactions to sensations (5). In recent years, research on the psychological effects of physical exercise has been strengthened (6). The number of interventions involving physical activity or movement in children and young people has increased, focusing on the importance and effect of physical activity on executive functioning (7), attention (8), and motor and cognitive skills (9).

Participation in physical activity affects levels of depression and anxiety, as well as mood levels (10) and contributes positively to the feeling of satisfaction with life (11). Physical involvement has been shown to facilitate social communication and, in a team context, group participation (12). From a cognitive point of view, numerous studies conducted in various countries have highlighted that physical exercise contributes to improving cognitive abilities both in healthy adults and in elderly people with pathologies, showing positive effects throughout life (13, 14). Interventions focused on motor skills have been shown to improve balance, coordination, postural stability (15–17) self-regulation, cognitive function (18), socialization (19), and verbal interaction abilities in children with ASD (20). In addition, engaging in aquatic-based physical activities has proven to be an effective approach for enhancing both motor abilities and social interaction skills (21–23).

Physical activity combined with music has been increasingly recognized as a promising approach for enhancing cognitive, social, and emotional outcomes, particularly in children with ASD. Music provides multisensory stimulation that can support attention, motivation, and emotional regulation, creating an enriched environment that enhances engagement during physical activity (24, 25). For children with ASD, music improves communication skills, social interaction, and emotional expression (26, 27), while physical exercise enhances motor coordination, executive function, and mood (4, 7). Rhythm-based interventions, in particular, have been shown to facilitate motor synchronization and social engagement by providing external temporal cues that enhance movement timing and coordination (28, 29). Combining these approaches may therefore synergistically promote improvements in both motor and cognitive domains.

Circadian rhythms—the body's intrinsic 24-h biological cycles—play a crucial role in regulating physiological and cognitive processes, including attention, memory, mood, and motor performance (30–37). Evidence suggests that cognitive performance and psychomotor abilities fluctuate throughout the day due to variations in hormone levels, core body temperature, and neural activity, influencing behavior, learning, and academic performance. The timing of physical activity may thus affect these outcomes (33), although findings remain inconsistent. Some studies suggest the morning is optimal for psychomotor performance (38), while others report benefits both in the morning and afternoon (39). Evening exercise has also been associated with improved performance (40) and combining physical exercise with cognitive training appears more effective in the afternoon (41, 42). Moreover, evening training may offer specific advantages for certain populations, such as older adults (43). For children with ASD, who often show differences in sensory processing and arousal regulation, aligning interventions with optimal circadian phases could further enhance benefits, but no study to date has examined whether the time of day of a movement and music intervention differentially affects cognitive function, mood, and social integration in this population.

This study aims to determine whether the time of day (morning vs. afternoon) modulates the effects of a combined movement and music intervention in children with ASD. We hypothesize that participation in such an intervention will lead to differential improvements in perceived competence and self-esteem, potentially influenced by the timing of the activity.

## 2 Material and methods

### 2.1 Participants

Firstly, 40 children (aged 6–10 years) with ASD were enrolled at the Jendouba Autism Centre (13 boys and 8 girls). Children with a diagnosis of autism spectrum disorder were enrolled based on DSM-V criteria (61). The diagnosis was confirmed through consultation with a therapist and a speech-language pathologist specializing in developmental disorders. Only children diagnosed with mild ASD (Level 1 or Level 2 support needs), without severe behavioral disturbances, were eligible for participation. Inclusion criteria were: (1) a confirmed diagnosis of ASD according to DSM-V standards; (2) absence of major behavioral issues likely to interfere with adherence to the program; (3) ability and motivation to engage in music- and movement-based sessions; and (4) absence of significant physical or medical conditions that could hinder safe participation. Exclusion criteria included significant verbal communication deficits, chronic illnesses, major comorbid disorders (e.g., epilepsy, severe intellectual disability), physical impairments, or injuries at the time of recruitment. A clinical assessment (CARS-2) was also performed to support ASD diagnosis and confirm symptom severity (44). These criteria ensured the selection of participants capable of engaging safely and effectively in the intervention program.

Based on the described selection criteria, a total of 30 children with ASD (19 boys, 11 girls; age 7.8 ± 1.27 years) were included in the study. Participants were randomly allocated into two intervention groups and one control group using dedicated randomization software designed to ensure an equitable and unbiased assignment. Randomization aimed to balance the groups in terms of age, gender, and ASD severity, ensuring baseline comparability. Precisely, the total of 30 children with ASD were divided into three groups. Twenty children participated in the intervention and were further divided into two subgroups based on the time of physical activity: the morning group (AM) (10 children: 7 boys, 3 girls) and the afternoon group (PM) (10 children: 7 boys, 3 girls). The remaining 10 children (6 boys, 4 girls) served as the control group (CG) and participated in their regular physical activity program at the Autism Centre.

Allocation concealment was maintained by an independent researcher who was not involved in participant recruitment or data collection. Group assignments were kept in sealed envelopes until the start of the intervention to prevent selection bias. Due to the nature of the intervention, participants and facilitators were not blinded to group allocation; however, outcome assessors and data analysts were blinded to group assignments to minimize detection and analysis bias.

The characteristics of the children are presented in Table 1.

**Table 1:** Children's characteristics.

Groups	Age (year)	Height (cm)	Body mass (kg)	Physical education experience (year)	Autism level
Morning group	7.8 ± 1.23	115.7 ± 3.2	24.9 ± 0.06	3.2 ± 0.63	1.5 ± 0.58
Afternoon group	7.9 ± 1.20	115.8 ± 3.1	25.3 ± 0.04	3.7 ± 1.06	1.3 ± 0.48
Control	7.7 ± 1.49	117.1 ± 3.3	25.8 ± 0.03	3.1 ± 1.37	1.2 ± 0.42

No participants were receiving medical treatment during the intervention. Children who had already received speech therapy or cognitive rehabilitation continued their usual care at a fixed time of one session per week. Medical stability was confirmed by families and physicians. All sessions followed a standardized protocol led by trained therapists.

Session fidelity was ensured through regular supervision, session checklists, and video recordings. Attendance and participation rates were recorded for all participants, with attendance above 95% in all groups. Reasons for any missed sessions (e.g., minor illness) were documented. No participants withdrew from the study; therefore, drop-out analysis was not applicable.

### 2.2 Ethical considerations

Ethical approval (approval reference: CPP N 13/2024, April 11, 2024) was granted by the Local Research Ethics Committee of the Higher Institute of Sports and Physical Education of Kef. The experimental protocol was conducted following the principles of the 2013 Helsinki Declaration. Parents were provided with detailed information about the study protocol and gave written consent for their child's participation, confirming their full understanding of the study's objectives, procedures, possible risks, and anticipated benefits.

### 2.3 Procedure

**Figure 1:**
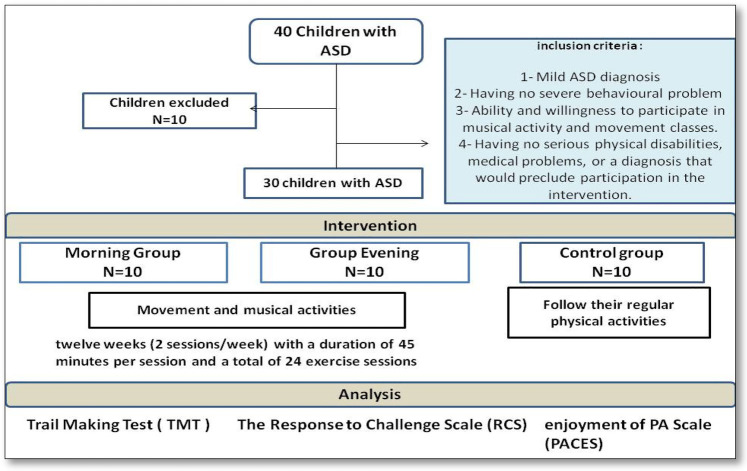
Study procedure.

The study was conducted from August to November. It lasted 14 weeks in total, including 1 week of pre-test (T1), 12 weeks of specific training (movement and music intervention), and 1 week of post-test (T2). The study aimed to examine the effect of the timing of sports practice on the cognitive functions, mood, and social integration of autistic children, using the Response to Challenge Scale (RCS), the Trail Making test (TMT), and also the Physical Activity Enjoyment Scale (PACES). Children were assessed before and after the intervention, using tests administered on 2 consecutive days, 48 h apart. All participants were familiarized with the study procedure, including the tests, before the initial assessment. During each session, children were assigned a 45-min work period, following the established protocol (Figure 1).

### 2.4 Intervention program

Children with ASD took part in a combined intervention program that included music, physical activity, and play-based motor tasks, as described in previous research (45). Specifically, twenty-four sessions, each lasting 45 min, were conducted over 12 weeks, with two sessions per week. The program was divided into three parts: (1) warm-up; (2) movement activities; and (3) musical activities.

The music used during the sessions consisted of rhythmic, child-appropriate songs with a regular tempo (90–120 beats per minute), selected to facilitate synchronization between auditory cues and body movements. Children were encouraged to perform movements such as walking, clapping, or jumping in time with the rhythm, promoting motor coordination, engagement, and enjoyment rather than using music as a passive background stimulus. This tempo range was chosen based on previous evidence suggesting that rhythmic auditory stimulation and tempo-structured musical cues effectively enhance motor synchronization, coordination, and movement fluency in children, including those with ASD (28, 29).

**Figure 2:**
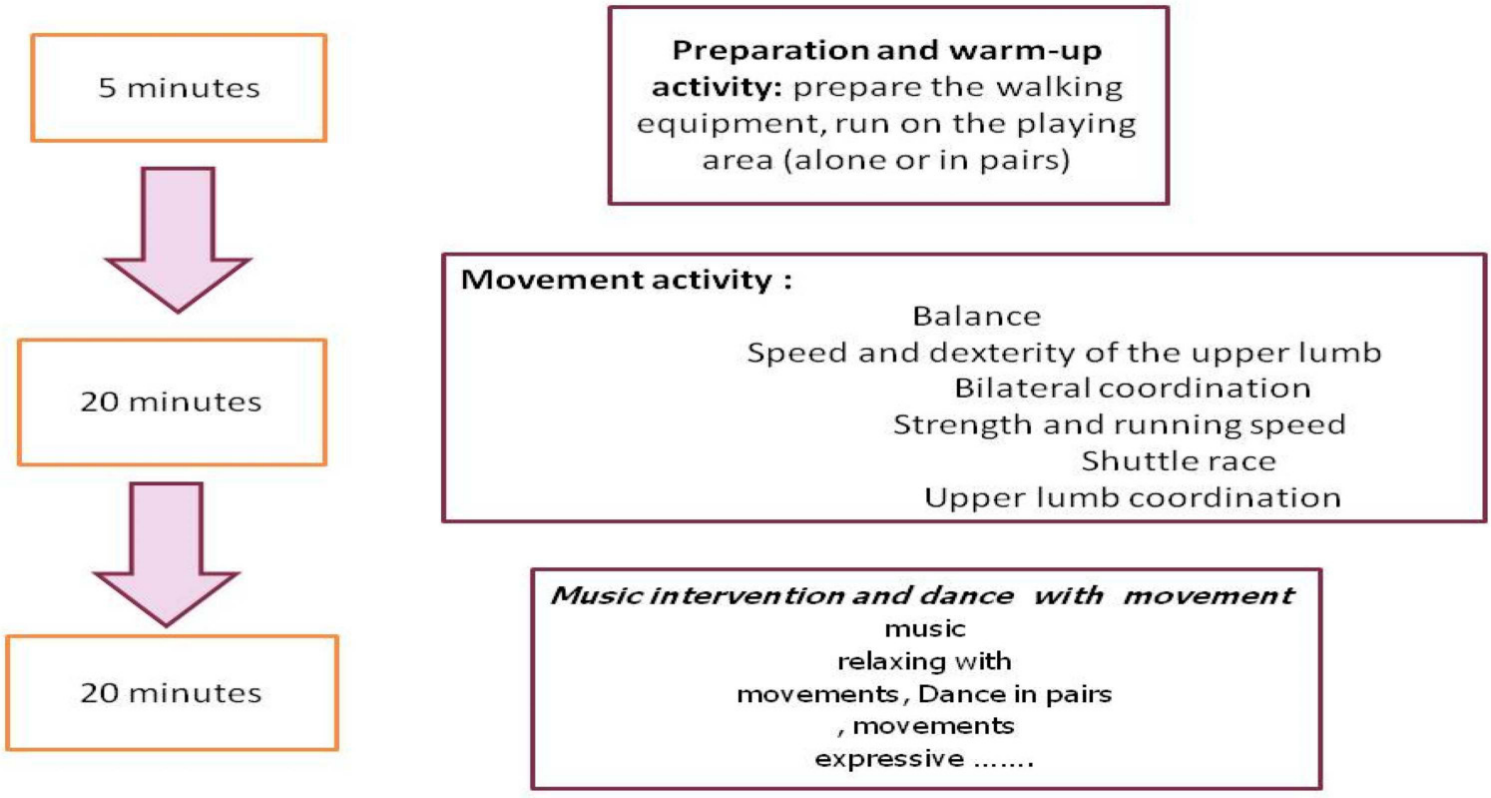
Intervention protocol.

Each session began with 5 min of warm-up, followed by 3 min of recovery, 20 min of movement activities, and concluded with 15 min of musical activities. The training protocols for the experimental group are outlined in Figure 2.

Specifically, one group participated in the activities in the morning (9:00–9:45 a.m.), while the second group participated in the afternoon (4:00–4:45 p.m.).

The control group did not undergo any structured intervention. They continued receiving the usual care provided at the ASD center, including services from occupational and speech therapists, and were encouraged to maintain their regular activities such as outdoor physical activity, fitness training, walking, running, and basic exercises. No specific intervention elements (e.g., music or creative tasks) were included in their program.

### 2.5 Measures

All children in the experimental and control groups performed, before and after the 12 weeks of intervention, in a randomized and counterbalanced order in the morning (9:00) and evening (16:00) the cognitive function test (TMT), the social integration test (PACES questionnaire), and the behavior problems test (RCS).

#### 2.5.1 Assessment of cognitive function

To examine intervention-related changes in cognitive function, the Trail Making Test was used (46), validated for children with neurotypical development and with ASD (47). This is a neuropsychological test created to assess flexibility mental, executive functioning, visual search speed, and calculation. It consists of two parts in which the subject must connect a sequence of 25 consecutive targets on a sheet of paper, with the test being used as the main measure. Of performance required to complete the test (for example, 35 s yielding a score of 35), lower scores are better.

#### 2.5.2 Assessment of social engagement and children's enjoyment

The enjoyment of physical activity was assessed by the PACES. This scale was designed to measure the positive effect associated with participation in physical activities in children and can be used for children with intellectual disabilities (48, 49) and with ASD (50).

The PACES questionnaire consists of 18 bipolar statements on a 7-point Likert scale (I like it—I hate it…), which are summed to give an enjoyment score. Each statement is designed to assess different dimensions of pleasure, offering a global vision of the child's attitude towards physical activity. Participants respond to each statement and their responses are then summed to obtain an overall rating of satisfaction. This score provides a valuable insight into how much children enjoy physical activity, which can influence their engagement, motivation and overall physical health.

#### 2.5.3 Assessment of stereotypical behavior

The Response to Challenge Scale (RCS) (33) was designed to measure self-regulation in children; it designed to measure cognitive, affective, and motor regulation skills in responses (51). The children here were asked to perform several tasks ranging from relatively simple (jogging in the field) to more difficult (jumping over a target Student). Performance on courses requiring three-dimensional of self-regulation skills such as Motor or physical control, Affective or emotional control and Cognitive control.

### 2.6 Statistical analysis

Statistical analyses were conducted using SPSS software (version 20.0; SPSS Inc., Chicago, IL, USA) for Windows. Data are reported as mean values accompanied by standard deviations. The assumption of normality was assessed using the Kolmogorov–Smirnov test. To evaluate differences between groups and over time, a univariate analysis of variance (ANOVA) was applied with the factors being group (experimental: morning vs. evening; and control) and time point [pre-intervention (T0) and post-intervention (T1)]. Diurnal variation effects were examined using a two-way repeated measures ANOVA, incorporating chronotype (morning or evening) as an additional factor. When significant baseline differences were identified, a two-way univariate analysis of covariance (ANCOVA) was used to adjust for those discrepancies. *post hoc* comparisons were carried out using the Bonferroni correction method in the presence of significant effects. The threshold for statistical significance was set at *p* < 0.05.

## 3 Results

### 3.1 Children enjoyment

**Figure 3:**
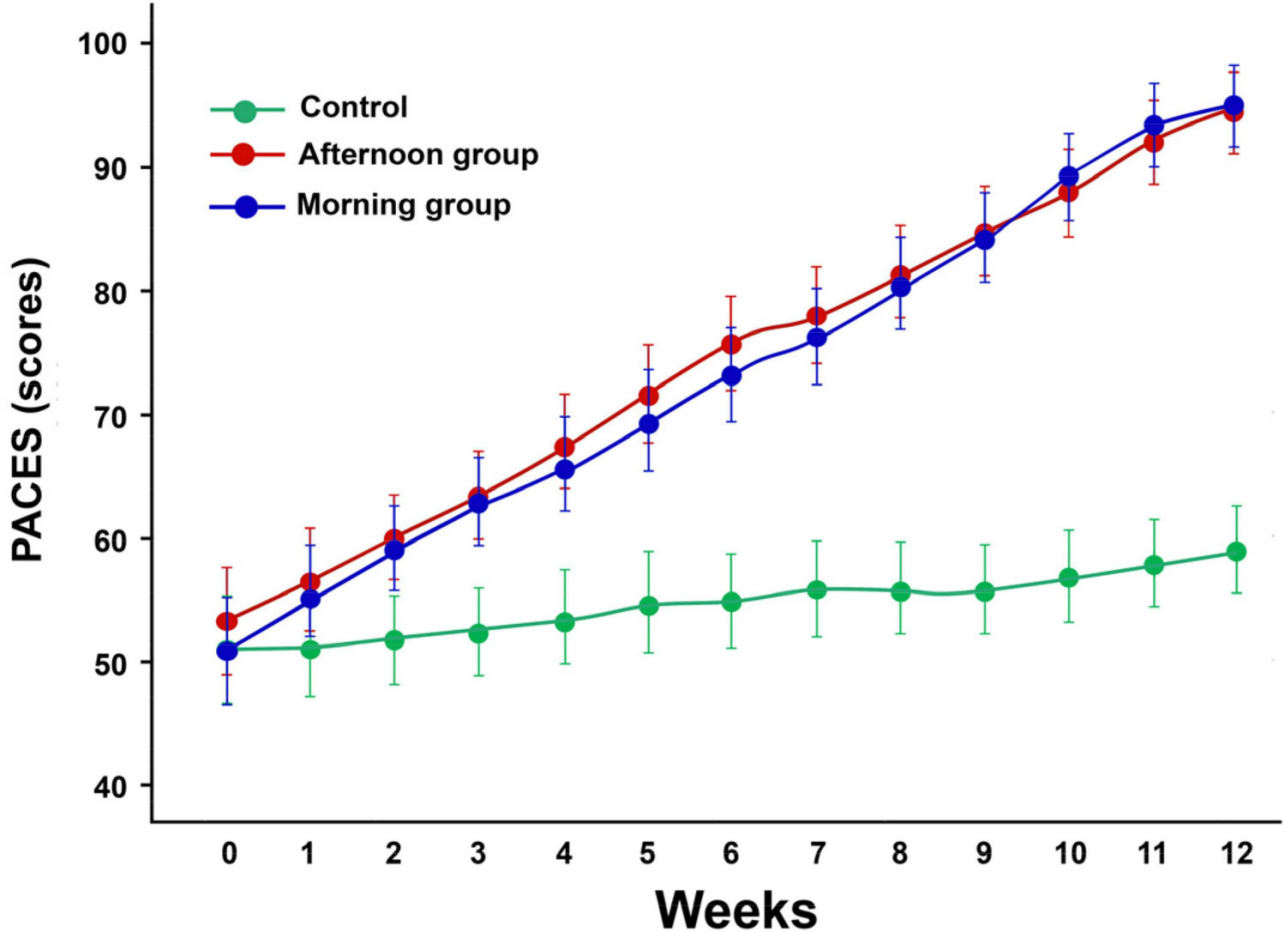
The mean scores of the physical activity enjoyment scale (PACES) valuated each week in both groups. Values are presented as means and standard deviations.

The PACES scale, designed to measure the positive effect related to children's participation in physical activity, was used to assess enjoyment. After 12 weeks of implemented music and movement intervention, the repeated measures ANOVA statistical analysis revealed a significant increase in participation and enjoyment (*F*_2_ = 77.826, *p* = 0.00001) in both experimental groups (morning and afternoon). PACES scores were likely very high in the experimental group (morning and evening) compared to the control group (*p* < 0.001) (Figure 3). Our results indicate that time of day (morning vs. afternoon) does not significantly influence the enjoyment of children. After intervention we do not find any difference between morning and afternoon) group (*p* = 0.743) (Figure 3).

### 3.2 Cognitive function

**Figure 4:**
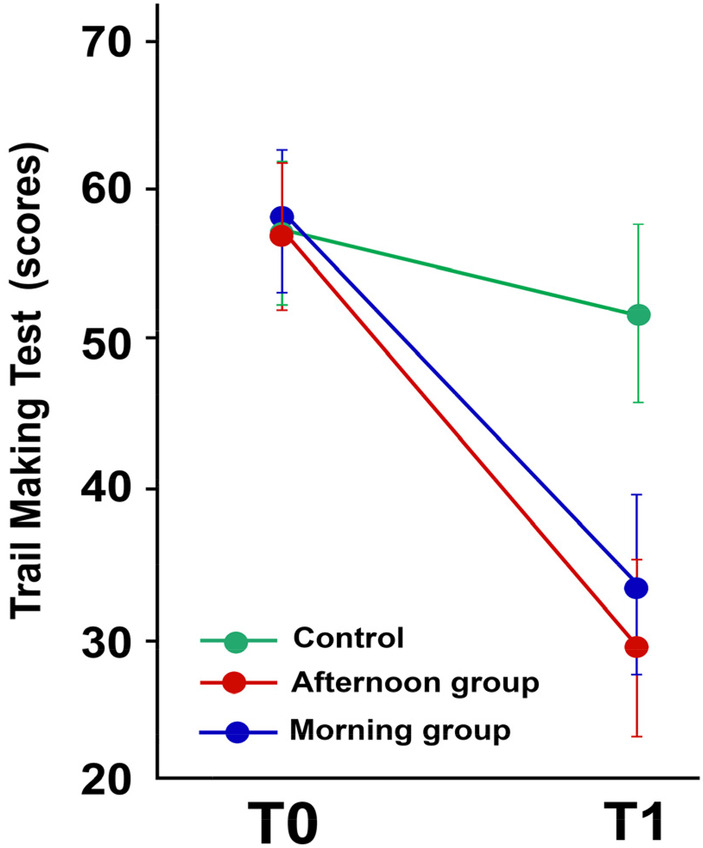
The mean scores of the trail making test (TMT) performed before and after the training program. Values are presented as means and standard deviations.

To evaluate the impact of the intervention on cognitive functions, the trail-making test was utilized. Figure 4 illustrates the cognitive function Scale (TMT) of both groups during their sessions, conducted before and after 12 weeks of intervention.

According to our results of a Repeated Measures ANOVA, statistical analysis revealed a significant group effect (*F*_2_ = 11,152, *p* = 0.00001). After 12 weeks of intervention/rehabilitation, the experimental group showed a significant improvement in scores compared to the control group (*p p* < 0.001). Additionally, scores were markedly higher in the experimental group (morning and evening) compared to the control group (*p* < 0.001). Similarly, the experimental groups demonstrated significantly improved scores at retest (*p* < 0.001).

### 3.3 Maladaptive behaviours

Table 2 presents the Response to Challenge Scale (RCS) values for both experimental groups—morning and evening groups—and the control group (CG) during their sessions before and after intervent. The scale is designed to measure motor/physical control, affective/emotional behaviours, and cognitive control.

**Table 2:** Average scores for stereotypical behaviours’ before and after the program training in both groups.

Stereotypical behaviours	Experimental group		
	Morning group	Afternoon group	Control group
Motors/physical control	14.59 ± 0.55	14.82 ± 0.56	10.47 ± 0.56
Affective or emotional Behaviors	35.29 ± 0.90	38.52 ± 0.89	26.80 ± 0.89
Cognitive control	32.55 ± 1.51	33.28 ± 1.51	23.62 ± 1.51
Total	82.43 ± 1.91	86.62 ± 1.91	60.89 ± 1.91

Means ± standard errors are shown.

Independent of the time of day, children in both the morning and evening groups showed a significant improvement in cognitive functioning compared to the control group. Additionally, children's memory performance was enhanced 24 h after training sessions in both groups. Overall, no significant difference was observed between the time of day and cognitive function in children with autism (*p* = 0.681).

After 12 weeks of intervention, statistical analysis revealed a significant group effect (*F*_2_ = 52.431, *p* < 0.001). The experimental group's results significantly improved after intervention training compared to the control group (*p* < 0.001). As shown in Table 2, an exhibited greater improvement in children's stereotypical behaviors was observed 24 h after both morning and evening chronotypes in motor behaviors among children with autism (*p* = 0.0001). However, our results indicate that time of day (morning vs. evening) does not significantly influence stereotypical behaviors in children with autism (*p* = 0.133).

Post-hoc comparisons revealed that for motors/physical control, both morning and afternoon groups significantly improved compared to the control group (*p* < 0.001), with no significant difference between morning and afternoon interventions (*p* = 0.45). For affective/emotional behaviours, both morning and afternoon groups improved significantly vs. the control group (*p* < 0.001), with no significant difference between the two experimental groups (*p* = 0.12). For cognitive control, both experimental groups outperformed the control group (*p* < 0.001), with no significant difference between morning and afternoon interventions (*p* = 0.58). Similarly, for the total score, both morning and afternoon groups showed significant improvements compared to the control group (*p* < 0.001), but no significant difference was observed between them (*p* = 0.33).

## 4 Discussion

This manuscript describes the effect of the time of day of physical activity and music intervention on cognitive function, mood, and social integration for children with autism. To our knowledge, several scientific studies have investigated the effect of physical activity in autistic children; however, no prior study has specifically examined the effect of time of day on cognitive function, social engagement, and maladaptive behaviors in this population. This study thus provides novel insights into the potential role of temporal factors in intervention design for children with ASD.

An important feature of our intervention is the combination of music with basic play movements, creating an engaging, multisensory experience. Several studies have highlighted the importance of physical training for children with ASD, noting its benefits for motor skills, emotional regulation, and adaptive behavior (8). Music, in turn, has been shown to enhance attention, arousal, and emotional processing (62). The integration of these elements in our program may therefore represent a synergistic approach, enhancing both motivation and the therapeutic effect.

The music and movement program used in the present study generated significant improvement in cognitive functions and specific stereotypical behaviors, while effects on broader maladaptive behaviors and social engagement were not significant in children with ASD.

These results align with prior research on children with ASD showing improvements in behavioral skills following physical activity programs (52) and music interventions (53). This suggests that targeted interventions combining both modalities may offer advantages over single-component programs, particularly in enhancing cognitive performance and reducing repetitive behaviors.

In our research, physical activity performed both in the morning and in the afternoon led to the same cognitive benefits, compared to the control group. After 12 weeks of intervention/rehabilitation, scores improved significantly in the experimental group in the experimental group across both time slots. This includes improvements in prospective memory, consistent with findings by Facer-Childs et al. (33), indicating that such benefits are robust regardless of circadian timing. This result aligns with literature indicating no clear advantage of a specific time of day for physical activity benefits on cognitive and behavioral outcomes (54–56).

However, some studies suggest time-of-day effects may vary depending on population and context. Takahashi et al. (42) reported that afternoon exercise improved cognitive function and mood in older adults, while studies involving children and adolescents have found variations in emotional state and behavior according to chronotype (57, 58). Our results show that, although time of day did not significantly alter cognitive outcomes in children with ASD, individual differences and contextual factors may still play a role. This highlights the need for personalized approaches in intervention planning.

Children in both groups consistently showed greater efficiency either in the morning or afternoon, suggesting that the intervention's effectiveness is robust across different circadian phases. Importantly, this finding has practical implications for educational and therapeutic planning: it suggests flexibility in scheduling music-based movement interventions without compromising effectiveness, which can facilitate integration into school and therapy programs.

Our findings are consistent with those of Kanzari et al. (45), who reported that a combined music- and movement-based intervention significantly improved motor competence, social engagement, and adaptive behavior in children with ASD. These results reinforce the relevance of multimodal approaches that engage both physical and auditory pathways to support neurodevelopment in ASD.

Although improvements in social engagement were not a primary outcome of this study, significant changes were observed in affective/emotional behaviours measured by the Response to Challenge Scale (RCS), suggesting potential indirect benefits for social engagement. This finding warrants further investigation using validated tools specifically designed to assess social engagement in ASD.

Overall, our findings suggest that, regardless of the time of day, participation in music-based movement interventions can improve cognitive functioning and reduce specific stereotypical behaviors in children with autism spectrum disorder, while effects on broader maladaptive behaviors and social engagement were not significant. These findings highlight the potential of such interventions as a complementary strategy in educational and therapeutic settings for children with ASD, supporting their inclusion in individualized care plans.

Despite the promising findings, several limitations should be acknowledged. First, the sample size was relatively small, which may limit the generalizability of the results to the broader population of children with autism spectrum disorder (ASD). Second, although randomization was applied, the groups may still differ in unmeasured variables such as home environment, prior exposure to music or physical activity, and individual preferences, which could have influenced the outcomes. In addition, the novelty of the music–movement sessions could have contributed to participant engagement and improvements, suggesting a potential placebo or novelty effect. Moreover, the cultural context of the intervention—including the type of music used and the specific group dynamics—may limit the generalizability of the findings to different cultural or educational settings. Furthermore, we acknowledge that the cultural context of the intervention—including the type of music used and the specific group dynamics—may limit the generalizability of the findings to different cultural or educational settings. Music preferences, cultural norms, and group interaction styles can vary widely, and these factors may influence both engagement and outcomes. Future studies should explore diverse cultural contexts and adapt interventions accordingly to enhance external validity.

Third, the intervention duration was limited to 12 weeks; longer interventions might be needed to assess the stability and sustainability of the observed benefits.

Third, the intervention duration was limited to 12 weeks; longer interventions might be needed to assess the stability and sustainability of the observed benefits. Fourth, although the time of day was considered, the actual circadian preferences (chronotypes) of the children were not assessed, which may have influenced their responsiveness to the intervention. Emerging evidence suggests that children with ASD often display a tendency toward similar chronotypes, with a higher prevalence of evening-oriented patterns compared to neurotypical peers. Studies have reported that a substantial proportion of children with ASD exhibit delayed sleep–wake patterns, with eveningness being significantly more common than in neurotypical populations (59, 60). Furthermore, older age in individuals with ASD has been associated with later chronotypes, greater social jetlag, and increased daytime sleepiness. This relative homogeneity in chronotype could reduce variability in responses to morning vs. afternoon interventions, partially explaining the lack of significant time-of-day effects observed in our study. In addition, time-of-day effects might also reflect contextual factors related to school scheduling, such as lesson timing, classroom routines, or general levels of alertness and engagement at different times of the day, rather than purely circadian biology. Future research should incorporate chronotype assessment and control for contextual variables to clarify their roles in optimizing intervention timing.

Finally, subgroup analyses based on gender, age, or baseline ASD severity were not performed due to the limited sample size. This may have masked potential differential responses to the intervention. Future research with larger cohorts should include such analyses to better understand how these factors influence intervention outcomes.

## 5 Conclusion

This study provides preliminary evidence that music-based movement interventions are feasible and may contribute to improvements in cognitive and behavioral outcomes in children with autism spectrum disorder (ASD). While no significant time-of-day effect (morning vs. afternoon) was observed, regular participation in structured physical activity appears to offer potential benefits for children with ASD. These findings may inform clinicians, educators, and rehabilitation specialists in designing engaging, non-pharmacological programs that support the developmental and emotional well-being of children with autism. However, larger and longer-term studies are needed to confirm these effects and strengthen the evidence base.

## Data Availability

The raw data supporting the conclusions of this article will be made available by the authors, without undue reservation.
